# An investigation of desalination by nanofiltration, reverse osmosis and integrated (hybrid NF/RO) membranes employed in brackish water treatment

**DOI:** 10.1186/s40201-017-0279-x

**Published:** 2017-07-21

**Authors:** M. Talaeipour, J. Nouri, A. H. Hassani, A. H. Mahvi

**Affiliations:** 1 Department of Environmental Sciences, Graduate School of the Environment and Energy, Science and Research Branch, Islamic Azad University, Tehran, Iran; 2 School of Public Health, Tehran University of Medical Sciences, Tehran, Iran; 3 Center for Solid Waste Research, Institute for Environmental Research, Tehran University of Medical Sciences, Tehran, Iran

**Keywords:** Desalination, Groundwater, Nanofiltration membrane, Reverse osmosis membrane, Hybrid (NF/RO), Reverse osmosis system analysis (ROSA) model

## Abstract

**Background:**

As an appropriate tool, membrane process is used for desalination of brackish water, in the production of drinking water. The present study aims to investigate desalination processes of brackish water of Qom Province in Iran.

**Methods:**

This study was carried out at the central laboratory of Water and Wastewater Company of the studied area. To this aim, membrane processes, including nanofiltration (NF) and reverse osmosis (RO), separately and also their hybrid process were applied. Moreover, water physical and chemical parameters, including salinity, total dissolved solids (TDS), electric conductivity (EC), Na^+1^ and Cl^−1^ were also measured. Afterward, the rejection percent of each parameter was investigated and compared using nanofiltration and reverse osmosis separately and also by their hybrid process. The treatment process was performed by Luna domestic desalination device, which its membrane was replaced by two NF90 and TW30 membranes for nanofiltration and reverse osmosis processes, respectively. All collected brackish water samples were fed through membranes NF90-2540, TW30-1821-100(RO) and Hybrid (NF/RO) which were installed on desalination household scale pilot (Luna water 100GPD). Then, to study the effects of pressure on permeable quality of membranes, the simulation software model ROSA was applied.

**Results:**

Results showed that percent of the salinity rejection was recorded as 50.21%; 72.82 and 78.56% in NF, RO and hybrid processes, respectively. During the study, in order to simulate the performance of nanofiltartion, reverse osmosis and hybrid by pressure drive, reverse osmosis system analysis (ROSA) model was applied. The experiments were conducted at performance three methods of desalination to remove physic-chemical parameters as percentage of rejections in the pilot plant are: in the NF system the salinity 50.21, TDS 43.41, EC 43.62, Cl 21.1, Na 36.15, and in the RO membrane the salinity 72.02, TDS 60.26, EC 60.33, Cl 43.08, Na 54.41. Also in case of the rejection in hybrid system of those parameters and ions included salinity 78.65, TDS 76.52, EC 76.42, Cl 63.95, and Na 70.91.

**Conclusions:**

Comparing rejection percent in three above-mentioned methods, it could be concluded that, in reverse osmosis process, ions and non-ion parameters rejection ability were rather better than nanofiltration process, and also better in hybrid compared to reverse osmosis process.

The results reported in this paper indicate that the integration of membrane nanofiltration with reverse osmosis (hybrid NF/RO) can be completed by each other probably to remove salinity, TDS, EC, Cl, and Na.

## Background

Water is of vital natural resources which is indispensable for economic, social and environmental sustainable development [1]. Drinking water quality including chemical and microbial standards and guidelines are designed to provide safe water for human consumption, thereby protecting public health [2]. Therefore, many studies conducted to present technologies for removal of organic matter [3, 4], removal of hazardous pollutants [5]. Other studies focused for introducing mathematical instrument for calculating the water quality index [6, 7]. Meanwhile, the water shortage is also considered and studied as important challenge of the current century which may result in several universal revolutions [1].

Climate changes and anthropogenic factors could influence on renewable water resources and water quality and quantity, as well. These water pollutants became resistant to usual water treatment methods, and affect adversely on the environment and human health [8]. Today, many regions throughout the world are faced with water shortage and crisis, due to several causes, like as rapid population growth, increasing demand, low precipitation, excessive exploitation of available water resources, as well as unequal distribution [9]. Regarding increasing demand for drinking water and a decrease of fresh water resources, use of desalination technology is of crucial importance for researchers. One of these technologies being applied for the preparation of drinking water from brackish water is known as Reverse Osmosis process [10]. Moreover, to remove the salts from brackish water and production of fresh water, desalination technology, particularly membrane process is used [11, 12].

The performance of membrane processes during separation of salts and ions from water is determined based on pores size and physical structure of different kind of membranes. Reverse osmosis membrane contains the smallest membrane pores. This small size of pores and reverse driven pressure causes a separation of water-soluble molecules. Nowadays, in addition to RO, Nanofiltration membrane is used especially in water treatment and water hardness removal, due to close similarity to RO [13].

Most of the countries which are facing with water shortage have been located in the Middle-East and northern Africa [9]. Due to desertification phenomena, reduction of precipitation, as well as drought during last 30 years, some parts of Iran, like as Qom province, suffer from water shortage [14]. No permanent river has been reported for the studied Province, located in the vicinity of the salt lake [15].

Khodadadi and, Mahvi et al., investigated Birjand city, located in the arid and semi-arid area, in order to produce drinking water from brackish groundwater using reverse osmosis process [16]. Another study, carried out by Mohebbi et al., the most appropriate method applied in water treatment in the hot and arid area, and highly satisfied by consumers, was RO process [17]. Some other researches were done elsewhere regarding the comparison of two NF and RO membrane and also hybrid and ROSA simulation model. Vaseghi et al., [18] also investigated rejection of Na^+1^ and Cl^−1^ ions and electric conductivity, as well using NF membrane and RO process at New Mexico University. They reported that in NF membrane and RO, rejection percent of Cl^−1^ ion was less than Na^+1^ and electric conductivity parameter [18]. In addition, Zhou et al. 2016 studied the desalination of Shanghai coastal waters, in China, using hybrid desalination method, in order to the preparation of drinking water [19].

An investigation was done by Naidu et al., [20], entitled “Comparison of Nanofiltration and Reverse osmosis processes in drinking water production from surface brackish water using 5 bar pressure and particular membranes, made in India [20]. In 2012, a study was done regarding water treatment using RO membrane and ROSA simulator, in order to compare rejection percent of total dissolved solid, at eastern Mediterranean [21]. Moreover, in this study ROSA software model was applied to simulate the increase of pressure influence on membrane performance.

The present study has been carried out in Qom province in Iran during 2015-16.

According to literature, researches have been done in overseas and Iran in subject of comparing function nonofiltration and reverse osmosis membranes [22, 23]. On the other hand, up to now, there is no research being reported on the comparison of three methods membranes (NF, RO, Hybrid NF/RO) by pilot and simulated the effect of increasing operation pressure on membranes function and hybrid system by ROSA software in arid- semi arid area.

The novelty of present research is the comparison between nanofiltration and reverse osmosis, and hybrid method. Also, besides the applied pilot plant, Rosa software was used to illustrate the effect of high operating pressure on membranes and hybrid system performance. Therefore, the main aim of present research is the performance evaluation of membrane in Qom water supply which is located in geostrategic region.

## Methods

### Case study

**Fig. 1:**
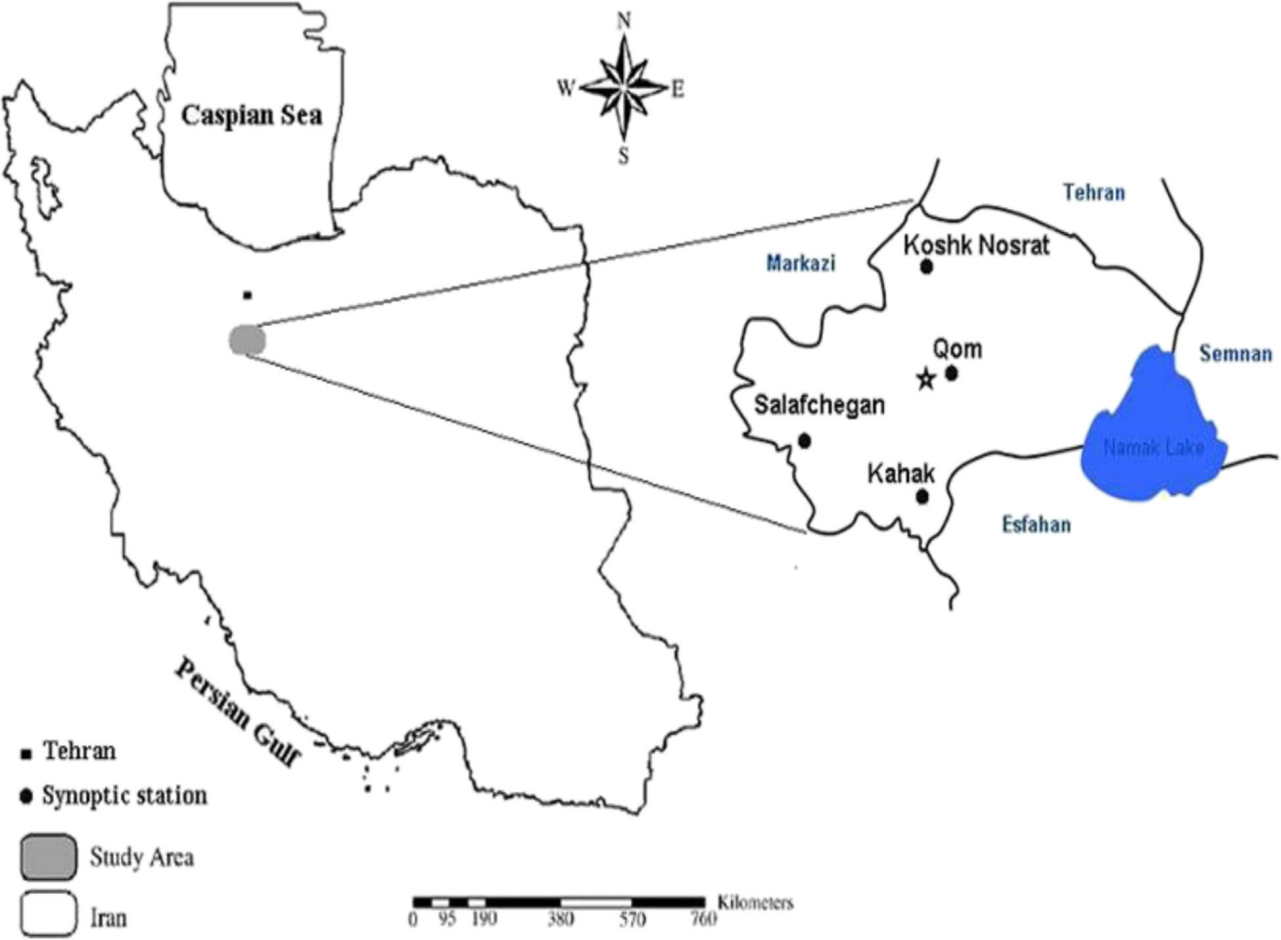
The study area of Qom province in Iran

The present study was carried out based on applied-descriptive studies, since beginning 2014 till beginning 2015 during 1 year in pilot form at central laboratory of Water and Wastewater Company of Qom province. The studied area is located in arid and semi- arid region, in Iran, stretched between 50° and 04′ to 51° and 03′ northern latitude and 34° and 27´ to 35° 12′ eastern longitude. Also, average precipitation was recorded less than 100 mm annually, so that, required water is usually supplied by groundwater, as well as water wells of Ali Abad. Due to vicinity by Salt Lake, groundwater in Qom Province contains salt and other dissolved solids. Hence, water quality is known to be inappropriate, with low quality of brackish waters in the study area [24]. The location of the study area is shown in Fig. 1. For desalination and treatment of brackish water, Luna fresh water device with 100GPD capacity, made in Luna Water Company of Canada, was used [25]. Also, to improve the efficiency and comparison of two membrane processes by Farayand Sazan-e-Mahab Company, representative of Luna Water Company, some changes were done in the mentioned-above device as follow:

### Experimental set- up

Replacement of RO membrane with TW30-1821 RO membrane and adding NF90-2540 to the device in order to implement Nanofiltartion process separately [26]. By these changes and adding ability connectivity, the device will be prepared to work in hybrid mode.

To do the investigation, after achieving the permit of Water and Wastewater Organization of Qom province, the pilot plant was installed at central laboratory of the organization, located in Qom province. Ali Abad water wells were used as feed water to study the performance of membranes separately, as well as in hybrid mode. Afterward, salinity, total dissolved solids, electric conductivity, and Na^+^ and Cl^−^ were measured during 1 year, from beginning 2014 to beginning 2015, regularly, each week using NF, RO, and hybrid processes as demonstrated in Table 1. To this aim, portable Multi-Parameter Meter, HQ40D 53,000, made by HACH Company of UK, was used to measure the mentioned parameters. In other side, to measure Na^+^ and Cl^−^, a Precision Titration class Telescoping Filling Tube-Standard Number DAKKS4760161, made by Brand GMBH Company of Germany, and FP7Jenway Industrial Flame Photometer, made by Bibby Scientific Ltd. of UK, were used, respectively.

**Table 1:** Changes and concentration of parameters after treatment using hybrid (NF/RO), nanofiltration (NF), reverse osmosis (RO)

Parameters	Internal	Hybrid	NF	RO	*P* value
		Output	Output	Output	
Salinity%	2.43 ± 0.17	0.3 ± 0.05	1.21 ± 0.08	0.68 ± 0.07	<0.001
TDS mg/L	3000.9 ± 129.1	704.6 ± 68.6	1698.2 ± 87.5	1192.5 ± 63.8	<0.001
ECμmohs/cm	4771.3 ± 202.7	1124.9 ± 80.2	2690 ± 142.2	1892.8 ± 101.5	<0.001
CL mg/L	1223.4 ± 54.6	451.5 ± 31.2	965.4 ± 48	696.4 ± 42.6	<0.001
NA mg/L	686.4 ± 35.3	199.7 ± 20.1	438.3 ± 26.6	312.9 ± 20.9	<0.001

### Software formula and statistics analytics

To investigate the effect of pressure on the reduction of concentration in salinity, TDS, EC, and Na^+1^ and Cl^−1^, ROSA software model was applied. ROSA software is a model, applied to design and manipulate RO and NF systems, innovated and designed by Nissan at 2005 [27] and [28]. To calculate rejection percent, Eq. 1 was used [26]:

1 $$ \begin{array}{l}\mathrm{Rejection}\ \left(\%\right)\\ {}\mathrm{Rejection}\left(\%\right)=\left(\mathrm{Concf}-\mathrm{Concp}\right)\times 100/\mathrm{Concf}\\ {}\mathrm{Rejection}\left(\%\right)=\mathrm{Rejection}\ \mathrm{percent}\\ {}\mathrm{Conc}\mathrm{f}=\mathrm{Filled}\kern0.5em \mathrm{water}\kern0.5em \mathrm{concentration}\\ {}\mathrm{Conc}=\mathrm{Produced}\kern0.5em \mathrm{water}\kern0.5em \mathrm{concentration}\end{array} $$

To descript data and standard deviation and demonstrate assessment accuracy, assurance distance of 95% was used. Also, paired t-test was applied to identification the treatment effect in each process, and to compare between three groups during a year, hybrid or LMM model, generalized from Repeated Measure ANOVA, was used. All statistical analysis was carried out using SPSS23 software.

## Results

Table 1 represents concentration of parameters, including salinity, total dissolved solids, electric conductivity and Na^+^ and Cl^−^ in feed water, as well as concentration changes of each parameter and ions by Nanofiltration, Reverse Osmosis and hybrid.

**Fig. 2:**
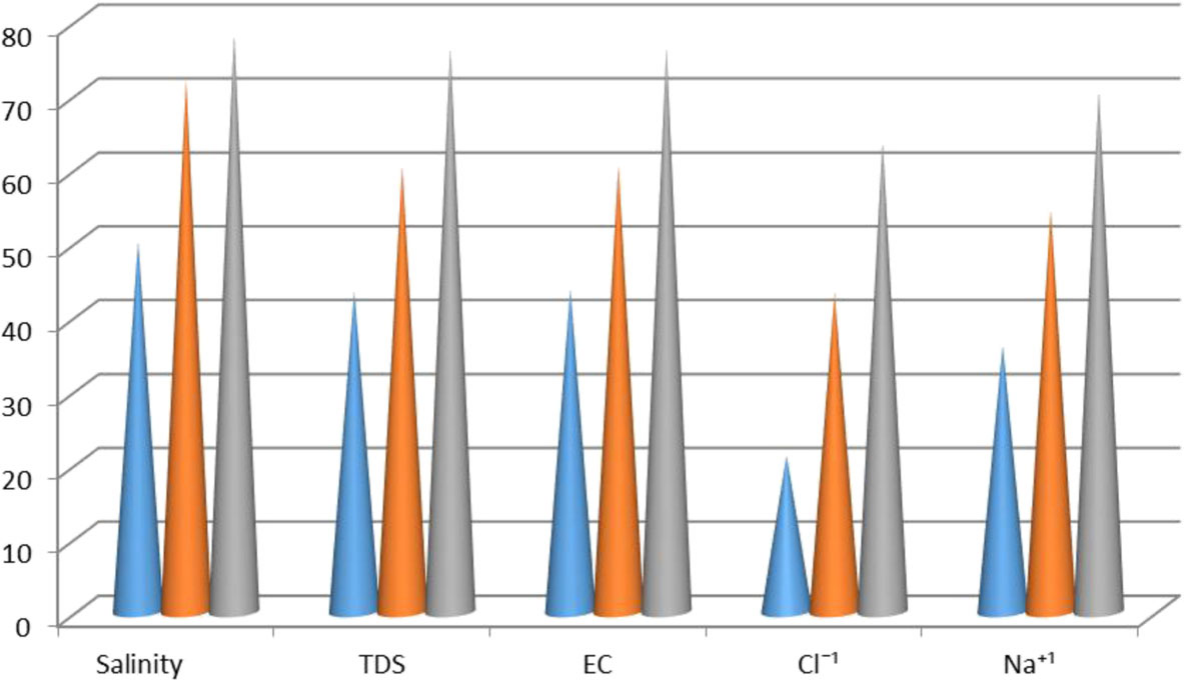
Rejection percent of studied parameters and ions using NF, RO and hybrid processes

According to Table 1, average salinity degree of feed water during 12 months was reported as 2.43 ± 0.17, reached to 1.21 ± 0.08, and 0.68 ± 0.02 and 0.3 ± 0.05%, being treated by NF, RO, and hybrid processes, respectively. Therefore, salinity rejection percent using NF, RO, and hybrid processes were measured 50.21; 72.01 and 87.65%, respectively. Average Total dissolved solids in the feed water, during 12 months of study, was 3000.9 ± 129.1 mg/L, reached to 1698.2 ± 78.5, 1192.5 ± 63.8 and 704 ± 68.6 mg/lit, treated using NF, RO, and hybrid processes, respectively. Hence, TDS rejection percent using NF, RO, and hybrid processes was recorded as 43.41, 60.26 and 76.52%, respectively. According to Table 1, EC in feed water during 12 months of investigation, was 1223.4 ± 54.6 μmohs/cm, reached to 2690 ± 142.1, 1892.8 ± 101.5,1124.3 ± 202.7 μmohs/cm being treated using NF, RO, and hybrid processes, respectively. So, EC rejection percent using NF, RO, and hybrid processes were calculated 43.62, 60.33 and 76.42%, respectively. Based on Table 1, average Cl^−1^, during 12 months, was measured in feed water 1223.4 ± 54.6 mg/L, reached to 965.4 ± 48; 696.4 ± 43.6 and 451.5 ± 31.2 mg/lit after treatment using Nanofiltration, reverse osmosis, and hybrid processes, respectively. Hence, rejection percent of Cl^−1^ using NF, RO and hybrid were 21.10, 43.8 and 63.95%, respectively. Based on Table 1, average Na^+1^, during 12 months, was studied in feed water 686.4 ± 35.3 mg/lit, reached to 438.3 ± 36.6; 312.9 ± 0.2 and 199.70 ± 20.10 mg/lit after treatment using Nanofiltration, Reverse osmosis, and hybrid processes, respectively. So, rejection percent of Na using NF, RO and hybrid were 36.15, 54.41 and 70.91%, respectively. According to mentioned-above Table, *P* value for all studied parameters and ions was *p* < 0.000 which demonstrates that reduction degree was significantly different in three mentioned methods. Table 2 represents rejection percent of studied parameters and ions using NF, RO, and hybrid processes. Figure 2 shows the percent of rejection for NF, RO, and hybrid processes.

**Table 2:** Rejection percent of studied parameters and ions using NF, RO and hybrid processes

Parameters	Rejection % NF process laboratory	Rejection % RO process laboratory	Rejection% Hybrid (NF/RO) process laboratory
Salinity	50.21	72.02	78.65
TDS	43.41	60.26	76.52
EC	43.62	60.33	76.42
Cl¯^1^	21.1	43.08	63.95
Na^+1^	36.15	54.41	70.91

**Fig. 3:**
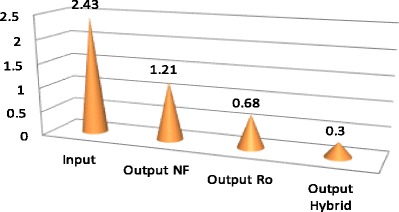
Variation of salinity (%) after desalination by NF, RO and Hybrid(NF/RO)

**Fig. 4:**
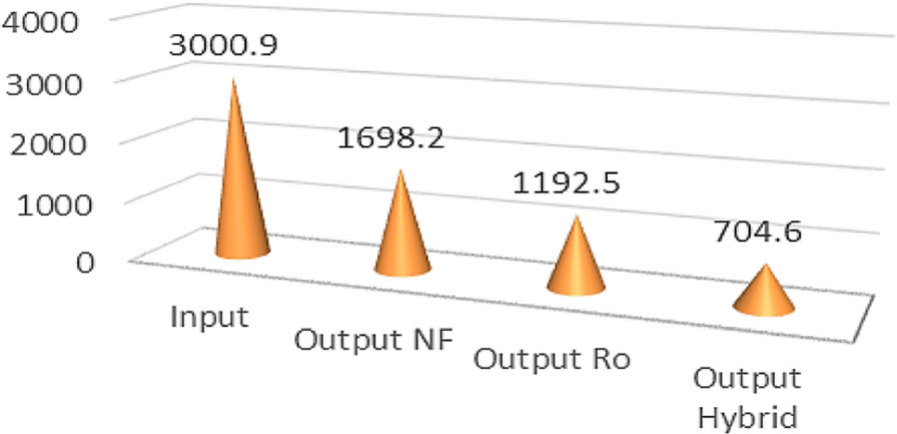
Variation of TDS (mg/L) after desalination NF, RO and Hybrid (NF/RO)

**Fig. 5:**
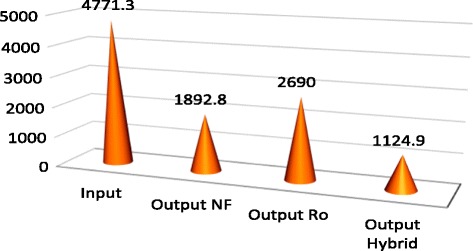
Variation of EC (μmohs/cm) after by NF, RO and hybrid (NF/RO)

**Fig. 6:**
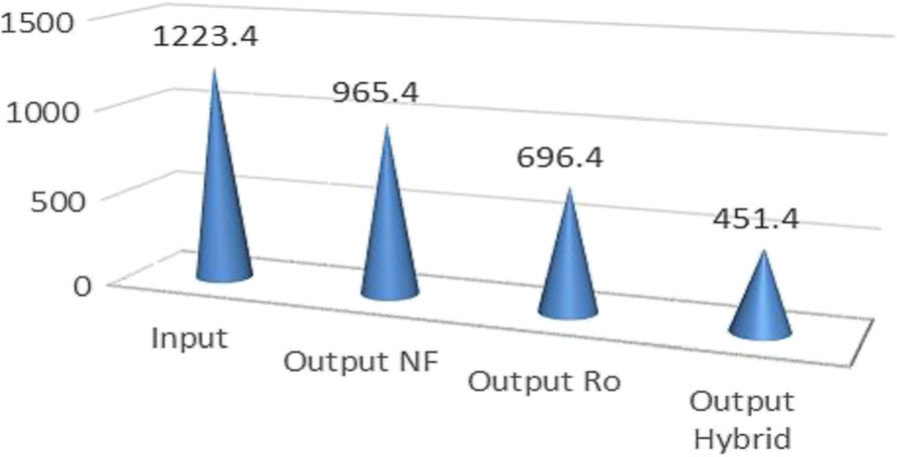
Variation of Cl¯(mg/L) after desalination by by NF, RO and hybrid (NF/RO)

**Fig. 7:**
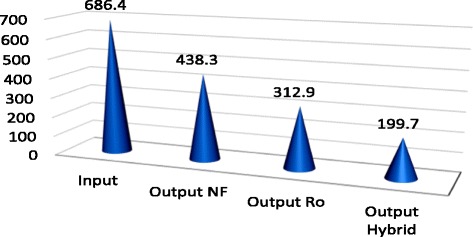
Variation of Na^+^ (mg/L) after desalination By NF, RO and hybrid (NF/RO)

Figures 3, 4, 5, 6 and 7 demonstrate very well variation of concentration in parameters, including salinity; Total dissolved solids; electric conductivity, as well as Na^+1^ and Cl¯^1^ using NF, RO and hybrid processes.

To investigate the relationship between higher pressure on concentration reduction in TDS and Na^+1^, Cl^−1^in feed water using NF, RO and hybrid processes, ROSA 8.0.3 software was applied. Results are shown in Tables 3, 4 and 5.

**Table 3:** Reverse osmosis system analysis for FILMTEC™ membranes ROSA 8.0.3 for NF membrane

Element type of membrane	Pressure driving force bar	
NF90-4040	6.96 bar	
Parameters mg/L	Internal (Feed water) mg/L	Output Permeable (NF Process) mg/L
TDS	3902.78	212.58
Cl¯^1^	1223.4	101.31
Na^+1^	812.41	67.53

**Table 4:** Reverse osmosis system analysis for FILMTEC™ membranes ROSA 8.0.3 for RO membrane

Element type of membrane	Pressure driving force bar	
BW30-4040	13.45 bar	
Parameters mg/L	Internal (Feed water) mg/L	Output Permeable (RO Process) mg/L
TDS	3838.81	29.01
Cl¯^1^	1223.4	10.52
Na^+1^	796.06	7.47

**Table 5:** Reverse osmosis system analysis for FILMTEC™ membranes ROSA 8.0.3 for HYBRID NF/RO

Element type of membrane	Pressure driving force bar	
HYBRID NF/RO	13.87 bar	
Parameters mg/L	Internal (Permeable NF as Feed water) mg/L	Output (Permeable) Hybrid (NF/RO) Process mg/
TDS	212.56	2.08
Cl¯^1^	101.31	0.8
Na^+1^	67.56	0.56

## Discussion

The salinity rejection percent was measured as 50.21%; 72.82 and 78.65% using NF, RO, and hybrid processes, respectively. Salinity reduction by Luna 100GPD, domestic fresh water device, using every three processes led to drinkable water. Results showed that rejection percent was higher in hybrid rather than RO and NF processes. During Nanofiltration and Reverse Osmosis processes, rejection percent was closely similar for TDS and EC parameters. In hybrid process, also, it was similar which declare that TDS and EC parameters are related to each other closely, as pointed out in a book entitled “Standard Methods for the Examination of Water and Wastewater, 2012” [29, 30]. Meanwhile, Salinity rejection percent was shown to be similar to TDS rejection percent, to some extent, which represents a direct relationship between salinity and TDS. In a research done by EPA, at 2015, this relationship between TDS and salinity parameters was noted [31]. Rejection of monovalent ions, such as Na^+1^ and Cl¯^1^ happens rarely in Nanofiltration and more usually in Reverse osmosis process. So, these mentioned ions are removed better in RO process rather than NF. However, rejection percent of Na^+1^ and Cl^−1^ using hybrid process is the best, in comparison with all two previous processes. Filmtec Company, in 2005, demonstrated that in Reverse Osmosis membrane, salinity, and TDS, as well as Monovalent ions parameters are removed better, compared to Nanofiltration membrane [26, 32]. Due to higher pressure in ROSA model, rejection percent of ions and studied parameters has been augmented regard to laboratory results. Consequently, higher pressure influences on concentration reduction and rejection percent of parameters, which leads to efficiency improvement of the membrane and produced water. Overall, in the hybrid process, difference degree of ions and parameters rejection percent in RO process was determined less than NF, and vice versa. Therefore, Nanofiltration process is more suitable to be applied as pre-treatment. To improve the results accuracy, findings and results of the present study have been compared with the other studies, carried out regarding the performance of NF, RO, and hybrid processes, elsewhere. Atab et al., [33] investigated a research entitled “Effect of desalination using Reverse Osmosis to produce Fresh water from brackish water: Case study: Naseriyeh River, located in Iraq”. They reported that augmenting the pressure from 25 bar to 63 bar, rejection percent of TDS reached to 99.8%. During the present study, increasing the pressure from 5 bar to 13.8 bar (in ROSA software model), rejection percent of Total dissolved solids changed to 99.2%, which is closely similar to Atab study [33, 34]. Naidu et al., [20], carried out a paper entitled” The comparison between Nanofiltartion and Reverse osmosis processes using Vadodara membrane, made in India, to produce healthy drinking water from groundwater”. The authors declared that rejection percent of Cl^−1^ ion, during %bar pressure was 65 and 72%, using NF and RO processes, respectively. In addition, rejection percent of TDS was 65 and 95%, in NF and RO processes, respectively. However, in the present study, Cl¯^1^ rejection percent was 21.1% in NF and 43.08% in RO process. Also, TDS rejection percent was measured as 43.41 and 60.26%, in NF and Ro processes, respectively. Due to use of different membranes in Naidu study, compared to the present study, results of rejection percent is different, which has influenced on mentioned parameters and ions rejection [20]. A research entitled “Desalination of offshore waters using hybrid (combined of Nanofiltration and Reverse osmosis) process, in Shanghai, China has been carried out in sciences Academy of Shanghai and laboratory of Marine Protection Technology by (Zhou et al., [19]). They reported that rejection percent of Total Dissolved Solids was 76.2% in the hybrid process, which is relatively similar to the present study, measured as 76.52% [19]. Another research was done by Kaya et al., [35] regarding treatment and desalination of offshore waters of Ezmir using Membrane processes, applying Nanofiltration membrane as reverse Osmosis pretreatment under 30 and 40 bar pressures. The authors understood that average rejection percent of salinity, TDS, EC and Na and Cl ions in hybrid process was augmented from 30 bar to 40 bar, under higher pressure. Similarly, throughout the present study, increasing the pressure from 5 bar to 13 bar using ROSA software model, rejection percent of studied parameters and ions were also augmented [35, 36]. In Arabia, at (2013), Ben-Meriem et al., investigated “desalination using Nanofiltration and reverse osmosis processes, as well as combined with two membranes in hybrid”. They measured salinity in NF, RO and hybrid process as 54.2%, 74.7%, and 83.3%, respectively. Thru the present study, also, rejection percent was reported 50.21 and 72.82, using NF and RO processes, respectively, which is seemed to be highly similar to Ben-Merrim study. Moreover, in a comparison, drawn between rejection of Na^+1^ and Cl^−1^, it has been demonstrated that rejection percent of Na^+1^ was higher than Cl^−1^ in both NF and RO processes. Similar results have been reported in the current study, within both membranes, alike to Ben-Meriem study [37]. Abhang et al., [38] studied the reduction of ions using Nanofiltration and Reverse osmosis process, in Puma University, India. They reported salinity rejection percent in both NF up to 50%, and 70-90 in RO processes. This study, measured salinity rejection percent using NF and RO as 50.21 and 72.82, respectively, closely similar to [38, 39]. In other hand, (Altaee, [21]) investigated water treatment with divers concentration using two Reverse Osmosis membranes throughout Eastern-Mediterranean. To do this, applied pressure and membrane kind were similar to pressure and membrane applied to ROSA model. Results showed that rejection percent of Na^+1^ and Cl^−1^, and Total dissolved Solids using RO membranes, was not significantly different from ROSA model. In the present study, due to higher pressure rather than software ROSA model, there reported some differences in rejection percent of Na^+1^ and Cl^−1^and TDS in Laboratory method. The main reason for equalities in results of Laboratory and software ROSA models was similar conditions of pressure and RO in both methods [21].

In Iran others studies have done about performance of RO that was able to decrease EC around range of 81.98%. Also another study declared that with increasing operation pressure removal of materials in water were increased [40, 41]. Other Researches of performance nanofiltration in Iran have done and revealed that NF membranes could decrease TDS in permeable very well if the concentration TDS in row water is around 3000 mg/L. Also Ions could be removed by applying nano filtration [42, 43]. In this research result showed that by increasing pressure in ROSA simulation the removal of parameters increased too and the TDS is in range of 3000 mg/lit could remove well and Ions by three methods of desalination.

## Conclusion

The salinity rejection percent using Nanofiltration, Reverse Osmosis, and hybrid processes were calculated as 50%; 70 and 74%, respectively. On this basis, rejection percent of Total dissolved solids; Electric conductivity and Na^+^ and Cl^−1^ ions parameters have been augmented in a hybrid process. Increasing the pressure, TDS, and Na^+1^ and Cl^−1^ concentration has been decreased in produced water significantly. Reduction rate, as well as rejection percent of TDS, EC and Na^+^ and Cl^−1^, was calculated to be higher in RO process rather than NF; and the highest, in a hybrid process. Also, Nanofiltration membrane plays pretreatment role for Reverse Osmosis membrane.

In summary, by comparison methods of desalination and hybrid system, find out hybrid system is the best for removing physical-chemical parameters such as salinity, TDS, EC, and ions (Cl and Na) in brackish water. Moreover, salinity and EC have the highest retention, then TDS. In addition, between the ions Na has been removed better than Cl in both membranes and hybrid system. In ROSA software result showed that by increasing operation pressure all parameters and ions have higher rejection than pilot plan in lower pressure. As Qom region located in semi-arid we recommend the commercial desalination plan will install by photovoltaic power operation instead of used electricity and utilize hybrid system in industrial membrane desalination in Qom.
